# There Is No Safe Dose of Prions

**DOI:** 10.1371/journal.pone.0023664

**Published:** 2011-08-15

**Authors:** Helen R. Fryer, Angela R. McLean

**Affiliations:** The Institute for Emerging Infections, Oxford Martin School, Department of Zoology, Oxford University, Oxford, United Kingdom; Nagasaki University Graduate School of Biomedical Sciences, Japan

## Abstract

Understanding the circumstances under which exposure to transmissible spongiform encephalopathies (TSEs) leads to infection is important for managing risks to public health. Based upon ideas in toxicology and radiology, it is plausible that exposure to harmful agents, including TSEs, is completely safe if the dose is low enough. However, the existence of a threshold, below which infection probability is zero has never been demonstrated experimentally. Here we explore this question by combining data and mathematical models that describe scrapie infections in mice following experimental challenge over a broad range of doses. We analyse data from 4338 mice inoculated at doses ranging over ten orders of magnitude. These data are compared to results from a within-host model in which prions accumulate according to a stochastic birth-death process. Crucially, this model assumes no threshold on the dose required for infection. Our data reveal that infection is possible at the very low dose of a 1000 fold dilution of the dose that infects half the challenged animals (ID50). Furthermore, the dose response curve closely matches that predicted by the model. These findings imply that there is no safe dose of prions and that assessments of the risk from low dose exposure are right to assume a linear relationship between dose and probability of infection. We also refine two common perceptions about TSE incubation periods: that their mean values decrease linearly with logarithmic decreases in dose and that they are highly reproducible between hosts. The model and data both show that the linear decrease in incubation period holds only for doses above the ID50. Furthermore, variability in incubation periods is greater than predicted by the model, not smaller. This result poses new questions about the sources of variability in prion incubation periods. It also provides insight into the limitations of the incubation period assay.

## Introduction

During the 1980s and 1990s, millions of Britons were orally exposed to bovine spongiform encephalopathy (BSE), yet fewer than 200 individuals have been diagnosed with variant Creutzfeldt–Jakob disease (vCJD). Understanding the circumstances under which exposure to transmissible spongiform encephalopathies (TSEs) leads to infection is important for managing risks to humans and animals from TSEs. With regard to the vCJD epidemic, it is clear that the species barrier was important in curtailing cases, but other mechanisms may also have played a role. For example, it is unclear how the probability of infection changes with the level of TSE exposure, especially at very low doses. Based upon ideas in toxicology and radiology [1] it is plausible that exposure to harmful agents, including TSEs, is completely safe at low enough doses [2], [3], but the existence of a threshold dose below which the probability of infection is zero is uncertain.

In the case of TSEs, there is some evidence to suggest such a threshold may exist. Mathematical models have been used to investigate the replication kinetics of prions – a general term for the proteinaceous etiological infectious agents of TSEs. It has been proposed that the smallest such infectious agent of TSEs is a polymer, made up of several monomers of PrP^Sc^ [4]–[6], the abnormal form of the prion protein. In that model the rapid conversion of the normal form of the prion protein (PrP^C^) to the abnormal form occurs only in the presence of polymers of a certain length. Polymers shorter than that length do not lead to rapid replication; instead they are unstable and can only grow to the required length through slow monomer addition. This mechanism was originally proposed to explain why spontaneous disease is so rare and yet disease appears to progress ‘inevitably after inoculation’ [6]. However, other evidence supports this mechanism. Namely it explains exponential accumulation of infectious material within a host [7], [8], prion strain diversity [9], [10], the linear appearance of fibrils [11], [12], some results from sonication experiments [13], and the failure to dissociate infectivity from aggregated forms of the prion protein [14]. A plausible consequence of this model is that there exists a threshold effect, such that if a host is infected with a concentration of PrP^Sc^ monomers that is lower than the critical polymer length then the infection does not propagate. Nevertheless the exact form or forms of the infectious agent of TSEs remains uncertain and so too does the mechanism of replication. Throughout this paper we use the term prion to describe a unit of the infectious agent that is capable of producing an infectious copy of itself.

We investigate the existence of a threshold infectious dose of prions using data from a very large collection of experiments (N = 127) in which mice were inoculated with varying doses of tissue from mice infected with mouse-passaged scrapie isolates. We also use these data to understand more about TSE incubation periods. The data includes records of whether each mouse became ill, and the incubation periods for those mice that developed symptoms. We compare these data to a simple stochastic model of prion replication. In the model the onset of disease is defined as the moment at which the number of prions reaches a predetermined limit [15]. Crucially, our model does not assume the existence of a threshold dose below which the probability of infection is zero. The three main questions that we address using the data are summarised here:

1. Is there evidence of a threshold dose of prions below which the probability of infection is zero?
2. How does mean incubation period change with dose; is it true that mean incubation period decreases linearly with logarithmic increase in dose?
3. How does the variation in incubation period change with dose; is it true that TSE incubation periods are highly reproducible?

To address the first of these questions we measure how the probability of infection in mice changes with dose. We show that infection is still possible at very low doses indeed, namely three orders of magnitude lower than the ID50 – the dose at which 50% of challenged hosts become infected. Furthermore the shape of this dose-response curve is consistent with the model of prion replication in which there exists no threshold dose of prions. Taken together these findings imply that there is no safe dose of prions. We find evidence to support the assumption of a linear relationship between dose and probability of infection in assessing the risk from low dose exposure.

In the remaining two questions we challenge two common assertions. The first is that mean incubation periods decrease linearly with logarithmic decrease in the dose of infectious material. The model predicts that the linear decrease in incubation period holds only for doses above the ID50; for doses below the ID50, incubation period is relatively invariant to dose. We observe precisely this pattern in the data. The second assertion is that incubation periods of prion diseases are highly reproducible. We measure the variability in incubation periods and compare it to model predictions. We find that although murine scrapie incubation periods appear very reproducible they are actually markedly more variable than predicted by the model. These findings have implications for prion studies that use incubation periods as a method to quantify the dose of an inoculum, emphasising that this method is only reliable at doses above the ID50. Furthermore they reveal a new perspective on prion incubation periods asking ‘why are they so variable?’ rather than ‘why are they so constant?’

## Results

### There is no safe dose of prions

To investigate evidence for the existence of a threshold dose of prions below which the probability of infection is zero we used a mathematical model and data collated from 127 different experiments in the murine scrapie model [16]–[21]. The experiments differed by mouse breed, tissue type, scrapie strain and route of inoculation, and within each experiment mice were infected at varying doses (10 fold dilutions). In total 4338 mice were inoculated. Incubation periods were recorded for those mice that showed clinical symptoms and the majority of mice were also tested post-mortem for pathological signs of scrapie.

First we analysed how the probability of infection changed with the dose of the inoculum. To enable us to meaningfully collate the data from different experiments we made use of the concept of the relative dose. This required us to calculate the ID50 for each experiment. However, for eight experiments it was not possible to estimate the ID50 with reasonable accuracy, thus data from these experiments (195 mice) were excluded from further analysis leaving 4143 mice to analyse. The rules used for the ID50 calculations are described in Figure S1. Once ID50s were estimated the ‘absolute dose’ within each experiment was then assigned a ‘relative dose’ – a relative dose of 0 is equal to the ID50, the positive integers are sequentially tenfold more concentrated than the ID50 and the negative integers, tenfold more dilute. More details of these experiments have been provided elsewhere [17]. In that study the data were also converted to the relative dose scale where it was shown that under this transformation there are no systematic trends that render the grouping of data from different experiments invalid.

**Figure 1:**
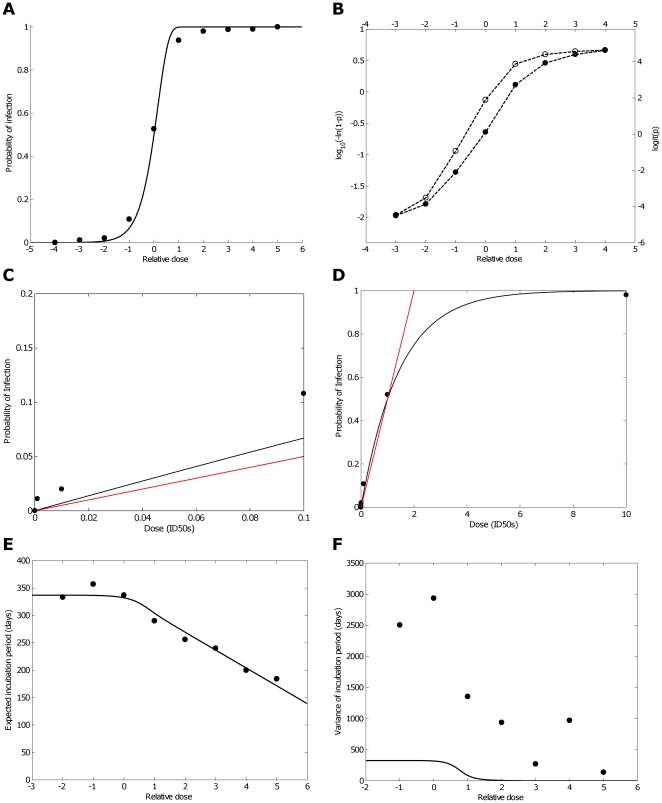
Data (circles) and model fits (solid black lines) showing how the proportion of mice infected and the expectation and variance of the incubation period vary according to the relative dose. A) The proportion of mice infected increases as the relative dose increases. The data reveals a sigmoidal pattern that fits well with the model that predicts that infection probability approaches zero at low doses and approaches one at high doses. In the data, infection probability was non zero as low as relative dose –3, but at relative doses –4 (the lowest dose tested) no mice were infected. However, the data at relative dose -3 and the model both indicate that a much larger sample size than the one used (N = 11) would be needed to find at least one infected mice at this dose. These results are consistent with the hypothesis that there exists no safe dose of prions. The modelled infection probability has no free parameters (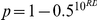) rendering the agreement between theory and data the more convincing. B) Two transformations are applied to the probability of infection data and the results are plotted against relative dose. Plotted as open circles is the transformation 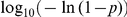. Using this transformation a straight line would indicate that the data are consistent with the model. Plotted as filled circles is the logit transformation, 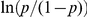, a function that is typically used to transform s-shaped data. Both transformations show similar results – plots that are close to linear from relative doses -2 to 1. Either side of these doses the data are almost linear, but less consistent with the model. Interestingly, at relative dose 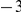 the probability of infection is slightly greater than predicted by the model. This is counter to what would be expected if infection probability was governed by a threshold dose. C and D) The probability of infection is plotted against dose in ID50s for low relative doses (C, ≤0.1 ID50s) and higher relative doses (D; ≤10 ID50s). These figures shows how the model assumes a linear relationship between dose and probability of infection at low doses (black lines). The data also supports the assertion that the probability of infection – whilst marginally greater than predicted by the model – is approximately linear at low doses. For comparison, the red line in each of these figures represents a linear relationship which necessarily has 0% probability at no dose and 50% probability of infection at the ID50. This comparison reveals that as dose increases, the relationship becomes increasingly less linear, particular beyond the ID50. E) The mean incubation period is dose-dependent. For relative doses above zero (the ID50), mean incubation period decreases linearly with the relative dose, whereas for relative doses below zero, it is relatively invariant to the relative dose. The model is fitted to the incubation period data using least squares to estimate two parameters. F) The observed variation in incubation periods is markedly greater than the variance predicted by the model. In this panel the circles show the observed variance of the difference from the group mean incubation period for mice from groups with at least two mice (Table 1, column 9). The black line represents model predictions of the variation of the incubation period. Since the net growth rate (*β-μ*) determines the maximum variance (see Figure 3C), this model prediction assumes that the net growth is equal to that estimated from the mean incubation period data shown in panel E.

Figure 1A and Table 1 show how the probability of infection changes according to relative dose when data from all of the murine experiments are collated. There is evidence of infection at very low doses indeed – three orders of magnitude below the ID50 (relative dose -3). At the lowest dose tested (relative dose -4), no mice were infected, however only eleven mice were challenged. Only one out of 92 mice tested positive at relative dose -3, indicating that the lack of infections observed at relative dose -4 could easily be explained by the small sample size. These data therefore do not provide any evidence that there exists a threshold dose below which the probability of infection is zero. Instead, they suggest that as dose decreases the probability of infection simply becomes smaller. Similarly, as dose increases the probability of infection simply increases. To demonstrate this under a more formal setting we compare the ‘dose-response’ data to predictions made by a very simple stochastic within-host model of prion propagation.

**Table 1:** Proportion of mice infected, mean incubation period and variance of incubation period at each relative dose.

Relative dose	Number of mice challenged	Number of mice infected			Proportion of mice infected	Mean incubation period	Variance of incubation period	Variance of difference from group mean incubation period (≥2 mice per group)
		In total	With an incubation period	In dose-experiment groups with ≥2 mice with an incubation period				
−4	11	0	0	0	0·000	-	-	-
−3	92	1	0	0	0·011	-	-	-
−2	294	6	5	0	0·020	333	3316	-
−1	591	64	59	38	0·108	357	25491	2503
0	712	375	352	330	0·527	337	17041	2935
1	644	604	589	584	0·938	290	10651	1354
2	461	452	439	439	0·980	256	9092	936
3	337	333	329	329	0·988	240	11041	270
4	207	205	203	203	0·990	200	7060	970
5	90	90	90	89	1·000	184	1329	136
**TOTAL**	**3439**	**2130**	**2066**	**2012**	**0**·**619**	**271**	**13174**	**1270**

At each relative dose the proportion infected (column 6) was estimated from all mice inoculated at that relative dose from 119 experiments for which an ID50 could be calculated. The mean (column 7) and variance (column 8) of the incubation period were estimated from all mice infected at that relative dose for which incubation period data were available (column 4). Part of the variability in incubation periods at each group is because of variability in the average incubation period between experiments. Column 9 shows the variance at each relative dose, excluding between-experiment variability in the average incubation periods. This metric was derived by first grouping mice according to experiment number and relative dose. For each mouse with an incubation period, we then calculated the difference between its incubation period and the mean incubation period of mice in that experiment-dose group. Data from experiment-dose groups for which there were fewer than two infected mice with an incubation period were excluded from this analysis (column 5). At each relative dose, the variance of these differences was then calculated (column 9).

**Figure 2:**
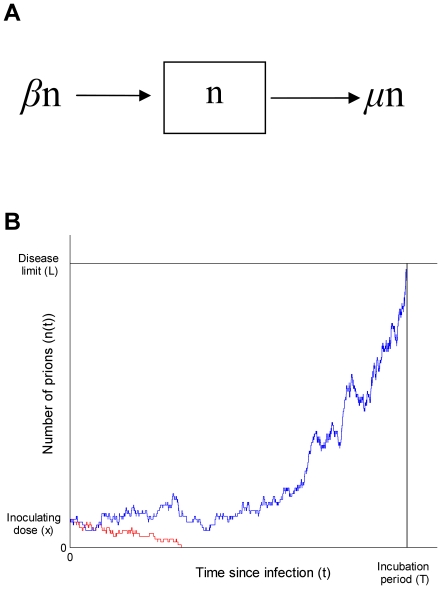
A stochastic model of prion replication. A) A mathematical model of prion replication in which the number of prions (*n*) changes according to a stochastic birth-death process. Prions are ‘born’ at rate *β* per prion and ‘die’ at μ per prion. B) In the model the onset of disease occurs once the number of prions reaches a predetermined ‘disease limit’ (*L*). An example of exponential prion growth up to the disease limit is shown by the blue line. Alternatively, by chance, the prion population can also become extinct and the host is left uninfected (red line). For both of these examples the same initial dose and model parameters are used. This model of a stochastic birth-death process with two absorbing barriers (at n(t) = 0) and n(t) = L) is a well-studied problem called the ‘Gambler Ruin’ [22].

In the model the host is inoculated with a certain number, *x*, of prions. Following inoculation, the total number of prions, *n*(*t*), at time *t*, changes because of two processes: prions can interact with uninfectious PrP^C^ monomers to create more prions at rate *β* per prion, or they can ‘die’ at rate μ per prion. Death could represent clearance of the prions and/or conformational change back to PrP^C^ monomers, but the process is immaterial since we assume no restriction on the availability of PrP^C^ monomers. Thus the replication dynamics of each prion is independent. This model is precisely a stochastic linear birth-death process as represented in Figure 2A. The onset of disease is assumed to occur once the number of prions has reached a predetermined ‘disease limit’ (Figure 2B black jagged line, n(*t*) = *L*) [15]. However, since this is a stochastic process that includes death of prions, the prion population can also become extinct (n(*t*) = 0), leaving the host uninfected (Figure 2B grey jagged line). If the model is considered for a fixed period, there is a third option – at cessation of the experiment the host is infected, but disease has not occurred. This possibility corresponds to the small number of mice with each dose that showed pathological lesions at post-mortem but had not showed any signs of disease. This model was chosen because it is the simplest system in which prion numbers grow exponentially [7], [8], the onset of disease is not certain, and infection probabilities are not governed by a dose threshold.

To compare the model to the dose-response data (Figure 1A, circles) we made use of the following expression that describes how the probability of infection, *p*, is expected to change with relative dose (RD): 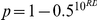. The derivation of this expression is provided in Text S1 [22], [23]. The analytic form and Figure 1A (black line) shows that the probability of infection changes sigmoidally with relative dose and is independent of other factors. For this curve there is no dose where the probability of infection is exactly zero or exactly one. Simply, the probability of infection tends to one as dose increases above the ID50 and tends to zero as dose decreases below the ID50. The model therefore provides an explanation of how the probability of infection can tail off dramatically at low doses in the absence of a threshold. The comparison in Figure 1A shows that the model fits the data well. Note that since the data is presented as a function of relative dose and since the expression for *p* is solely dependent upon relative dose, the data and model are compared directly without the need to fit any parameters. The model also suggests that it is unsurprising that no mice were infected at relative dose –4. With a predicted probability of infection of 7×10^−5^, a sample size of 14000 mice would be required to find only one positive. Only 11 mice were tested at that dose.

To compare the model and observations further, we transformed the infection probability data according to the function 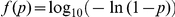 and plotted it against relative dose (open circles, Figure 1B). If the data were exactly consistent with the model this transformation would transform the data to a straight line. On this scale the data are close to linear from relative doses–2 to 1, but either side of these doses the data are less consistent with the model. It is noteworthy that where this happens at relative dose –3 the probability of infection is slightly *greater* than predicted by the model. This is counter to what would be expected if infection probability was governed by a threshold dose. For completeness, we have also transformed the data according to the logit transformation, ln(p/(1-p)) (filled circles, Figure 1B), a function that is typically used to transform sigmoidal data. This transformation shows a similar pattern. In summary, by comparing the murine data to the model, we find no evidence that there exists a safe dose of prions.

In risk assessments of exposure to TSE infectivity it is important to understand the relationship between dose and the probability of infection. The relationship can be used to translate exposure levels into predicted number of infections. Many such studies assume, either implicitly or explicitly, that the relationship between dose and infection probability is linear, i.e. doubling the dose leads to double the infection probability [24]–[28]. In other studies, non-linear relationships have been explored [29], [30]. In the context of TSE risk assessments it is often appropriate to assume that, even if many hosts are exposed, each host has only a low level of exposure. Figures 1C and 1D show the dose response curve replotted as a function of the dose in terms of number of ID50′s. These figures reveal that the model relationship between dose and probability of infection is linear at low doses (black lines). This can be confirmed mathematically (Text S1, expression 12) [23] and is intuitively obvious given the underlying assumption of our model that the replication dynamics of each prion is independent. The data also supports the assertion that the probability of infection – whilst marginally greater than predicted by the model – is approximately linear at low doses. For comparison, the red line in each of these figures represents a linear relationship which necessarily has 0% probability at no dose and 50% probability of infection at the ID50. This comparison highlights that that as dose increases, the observed relationship becomes increasingly less linear, particular beyond the ID50. In summary, for low dose exposure we find evidence in support of risk assessments assuming a linear relationship between dose and infection probability. Thus, doubling the dose leads to double the infection probability. As dose increases this relationship breaks down, particularly above the ID50. Use of the linear relationship at these higher doses is likely to overestimate, rather than underestimate, risk.

### Incubation period decreases linearly with relative dose only for doses above the ID50

There has been a long understanding that TSE incubation periods decrease approximately linearly with every tenfold increase in dose and are highly reproducible between hosts [6], [17], [31]. As such, incubation periods have been used in diagnostic tests as a proxy for dose [32], [33]. Deepening our understanding of TSE incubation periods therefore has important implications for ensuring accurate diagnostic tests. It may also lead to clues about prion replication mechanisms.

**Figure 3:**
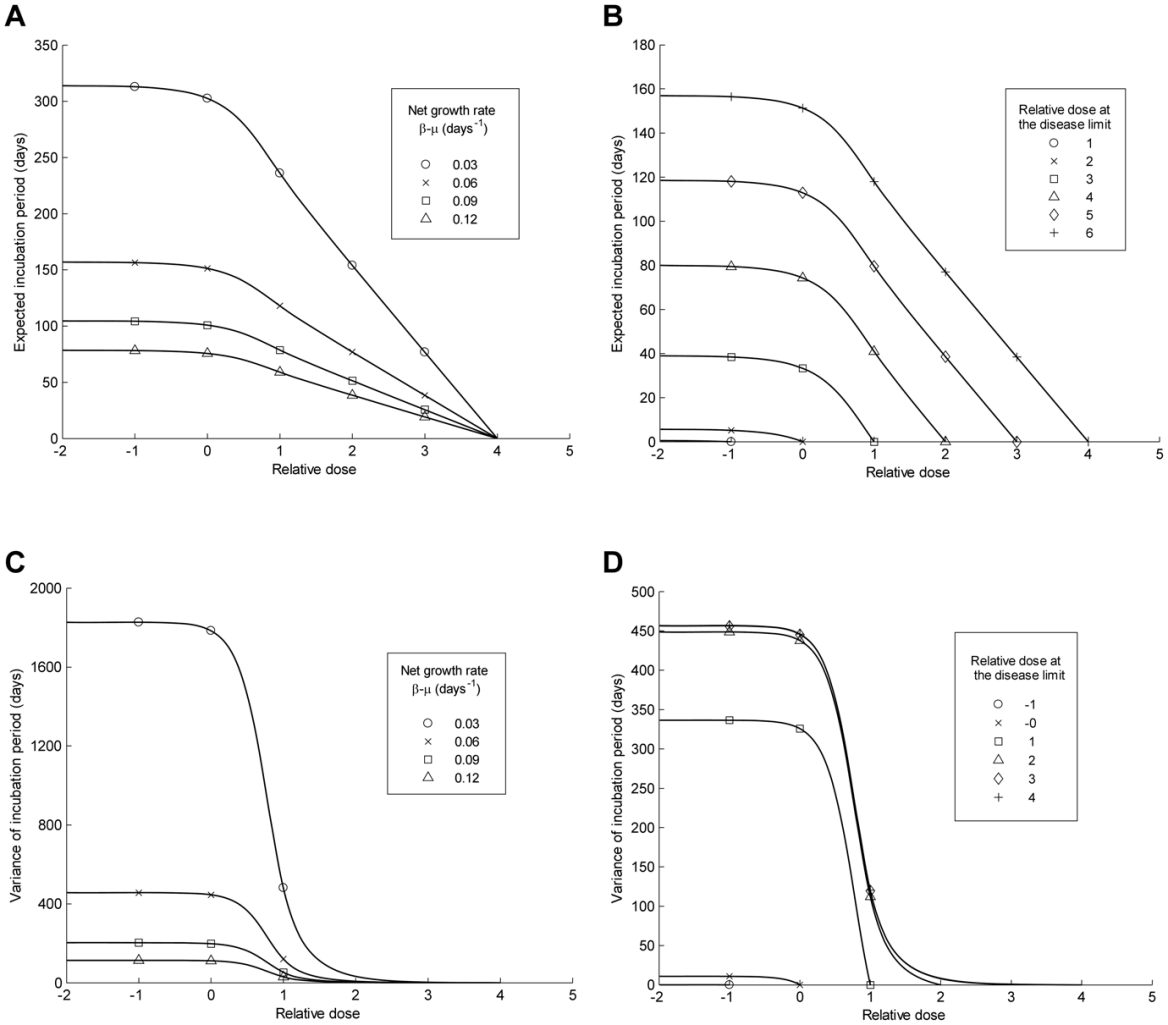
Model predictions showing how the expectation and variance of the incubation period vary according to the relative dose (RD), the net growth rate (β-μ) and the relative dose at the disease limit (RD_L_). A) and B) show factors affecting the expectation of the incubation period. The expected incubation period is approximately invariant to the inoculating dose for relative doses less than approximately 0 (the ID50). Beyond approximately 0, the incubation period decreases linearly with relative dose. A) shows that the gradient of this slope is steeper if the net growth rate of prions (*β-μ*) is smaller. Specifically the gradient is equal to 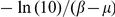. B) shows that the expectation of the incubation period is also larger if the relative dose at the disease limit is larger. C) and D) show factors affecting the variance of the incubation period. The variance is approximately invariant to dose for doses less than relative dose 0. Beyond 0 the variance decreases as the dose increases. The variance also increases as the relative dose at the disease limit increases, but has an upper bound that is reached when the relative dose at the disease limit is greater than 3. Thus the maximum variance is determined by the net growth rate (β-μ). In panels A) and C) the relative dose at the disease limit is assumed to equal 4. In panels B) and D) the net growth rate is assumed to equal 0.06 days^−1^. We also assumed that at relative dose –2 there was 1 prion in the population. This assumption truncates the graphs at relative dose –2, but does not change the shape of the graphs. The data included in this figure are from 119 experiments for which an ID50 could be calculated.

We have used the model and murine data to investigate how mean incubation periods change with dose. The deterministic form of the model (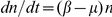) predicts that prion numbers grow exponentially (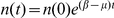) and therefore for all doses, incubation period should decrease linearly with logarithmic increase in dose, i.e. decrease linearly with relative dose. Each unit increase in relative dose should lead to a reduction of the incubation period by a value of 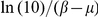, where 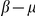 is the net growth rate of prions. At high doses this is what we see (Figure 1E). However, prion replication is a stochastic process and at low doses this stochasticity disrupts the dose-incubation period relationship. At doses below the ID50 the incubation period is predicted to be invariant to dose (Figures 3A and 3B) and this prediction is fulfilled by the data (Figure 1E). Why is this so? At doses below the ID50 more than half of the challenges fail to establish an infection. In effect the incubation period data is heavily censored to remove those individuals in whom the challenge dose goes extinct. The challenges which, just by chance, have slow initial growth are more likely to go extinct, or if they do not will produce the largest incubation periods. Thus at low doses the extinction process in the stochastic birth-death process censors some very long incubation periods and disrupts the linear relationship between dose and incubation period. At doses above the ID50, most hosts become infected, thus censorship has less effect and incubation periods correspond to those predicted by the deterministic model – they decrease linearly with relative dose. Figure 3A shows that the gradient of this part of the graph is inversely proportion to the growth rate of prions. Figure 3B shows that the expectation of the incubation period also increases as the relative dose at the disease limit increases. Specifically, beyond relative dose 0 the expectation of the incubation period is given by: 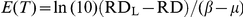. The derivation for this expression and the expression (Table S1), used to plot Figures 3A and 3B, which describes how the expectation of the incubation period changes with dose for all doses, is provided in Text S1 [34].

For those mice for which incubation period data were available (N = 2086) we grouped the mice according to relative dose (Table 1, column 4) and calculated the mean incubation period at each dose (Table 1, column 7). These data are plotted in Figure 1E and suggest that, as the model predicts, incubation period remains invariant to dose below approximately relative dose 0. Above relative dose 0 the data also follows the model prediction that the average incubation period decreases linearly with relative dose. Figure 1E shows the best fit of the model to the data. Least squares is used for this calculation. Only two values need to be fitted: the net growth rate of prions (*β*-*μ* = 0·07 days^−1^) and the relative dose at the disease limit (RD_L_ = 10·3). In summary, the model predicts that incubation period is invariant to dose at relative doses lower than the ID50, but decreases linearly with relative dose at doses above the ID50. The available murine scrapie data agrees with these predictions.

### Incubation periods are not as reproducible as predicted by the model

Since TSE incubation periods are regarded as being highly reproducible [6], we addressed the question: how does the observed variation in incubation periods compare to the variation predicted by the stochastic birth-death model. We derived an analytic expression to describe how the variance of the incubation period changes according to absolute dose (Table S1 and Text S1). In Figures 3C and 3D these predictions are plotted on the relative dose scale where they show that, like the expectation of the incubation period, variance is dependent only upon the relative dose (RD), the net growth rate (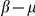), and the relative dose at the disease limit (RD_L_). Variance is invariant to dose for doses below approximately the ID50, and decreases with dose above the ID50. The variance is also larger if the relative dose at the disease limit is larger (Figure 3D). However, if the dose at the disease limit is 1000 fold or more greater than the ID50 (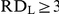) then variance does not increase any further. From the incubation period data we estimate that 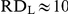, implying that the expected variance will be a function only of the net prion growth rate (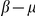) (Figure 3C). As described previously, this rate can be estimated from the mean incubation period data.

We used the model and our estimate of the net growth rate (0.07 days^−1^) to predict the variance of the murine incubation periods at different relative doses. This prediction is shown in Figure 1F (black line). To compare it to the variance observed in the mice, we considered two methods for evaluating variance. We first grouped mice from all experiments according to relative dose and then simply calculated the variance at each relative dose (Table 1, column 8). At each dose the observed variance was considerably greater than predicted. For example, at relative dose 0, the predicted variance was 320 days, yet the observed variance was 17000 days.

To understand whether the high level of observed variability was generated by variability between experiments, our second calculation aimed to eliminate such inter-experiment variability. For each dose within each experiment we first calculated the mean incubation period. For dose-experiment ‘groups’ with two or more infected mice with incubation period data (N = 2032) for each mouse we then calculated the difference between their incubation period and the mean for their group. The ‘differences’ from each experiment were then grouped according to relative dose and the variance of these differences at each dose was calculated (Table 1, column 9). These data are plotted in Figure 1F (circles). As expected, they show that variability in incubation period decreases with dose for doses beyond the ID50. They also hint that variance may, as predicted, stabilise at doses lower than the ID50 – variance is lower at relative dose –1 than at 0 – though there is no clear pattern. However, the most striking observation is that the observed variability is still much greater than the maximum predicted variance, particularly so at low relative doses. For example, at relative dose 0 the observed and predicted variances were 2900 days and 320 days respectively. This difference cannot be attributed to differential net growth rates between experiments. When the six largest experiments are each considered individually and net growth rates are calculated for each, the observed variance is still much greater than predicted (Figure S2). In summary we find that variability in incubation periods is much greater than predicted by a simple stochastic birth-death process, even though that model fits the data on infection probability and mean incubation period rather well. Thus, although incubation periods for prion diseases are remarkably reproducible in comparison with other infections, they are nevertheless substantially less reliable than if governed by a stochastic birth-death process.

#### The incubation period assay

Since end point titrations take a long time and require large numbers of mice, a technique called the ‘incubation period assay’ is now often used as an alternative method for determining the TSE infectious doses [32], [33]. In this technique, end point titrations are first used to calibrate the relationship between dose and incubation period. Incubation period measurements are then compared to this relationship to determine the dose of a sample, thereby saving both time and resources. Our study provides new insight into the relationship between dose and incubation period and therefore into the capabilities, or rather limitations, of the incubation period assay.

Our results imply that both the mean (Figure 1E) and variance of the incubation period (Figure 1F) is invariant to dose for doses below the ID50. This suggests that the incubation period assay cannot be used to distinguish between different infectious doses below the ID50. Consider, for example, one host infected with a relative dose of –1 and one with a relative dose of –2. We predict that both the mean and the variability of the incubation period at each of these doses would be the same. Thus, incubation period measurements from these hosts would provide no information to distinguish between their infectious doses. We note, however, that the incubation period assay should be capable of distinguishing – with a level of certainty linked to the abundance and spread of data used for calibration – between different doses above the ID50.

## Discussion

In this study we first asked whether there exists a threshold dose of prions below which the probability of infection is zero. By comparing the scrapie dose-response curve observed in mice to model predictions we found no evidence that such a threshold exists. As the stochastic-birth death model predicts, the probability of infection simply becomes smaller as the dose decreases. Furthermore, we find evidence to support the assumption of a linear relationship between dose and probability of infection in assessing the risk from low dose exposure. Use of a linear relation for doses above the ID50 will lead to overestimation of the risk.

Although we find no evidence of a threshold, it must be emphasized that we cannot rule out this possibility. Though it was to be expected because of the limited sample size, no mice were infected at the lowest relative dose tested (–4), therefore a threshold may exist at this dose or lower. However, acquiring data to investigate this question further would require an unfeasibly large number of test animals. Furthermore, the observation of infection at a dose 1000 times more dilute than the ID50 shows that infection is still possible at very low doses. In practical terms this is low enough to regard there to be no safe dose.

Previous modelling work has focussed on understanding the molecular form of a prion and its mechanism of replication. We are not proposing a specific form or replication mechanism, rather we ask whether data on infection probabilities are consistent with the simplest model of replication that assumes no threshold dose. If evidence did emerge of a threshold dose, one could tie it to the hypothesis that the smallest infectious agent involved in TSEs (a prion) is a polymer consisting of multiple PrP^Sc^ monomers above a critical polymer length [4]-[6] . It is thought that such a polymer replicates when it breaks into two or more polymers, each larger than the critical length, before undergoing rapid monomer addition. This is currently the most widely accepted mechanism of prion replication. However, it is noteworthy that a threshold polymer length is not the same as a threshold dose of prions, since a prion is normally regarded as one infectious agent (i.e. a polymer), not one PrP^Sc^ monomer. Dilution of infectious substrate would not necessarily split up prion polymers into units lower than the critical polymer length, thus, the polymer breakage-addition model of prion replication is consistent with our finding of no threshold for infectiousness.

In this study we also asked how TSE incubation periods change according to the inoculating dose. First, we asked whether it is true that mean incubation period decreases linearly with logarithmic decrease in dose. The model predicts that this relationship holds only at doses higher than the ID50. For doses below the ID50, mean incubation period is predicted to be invariant to dose. The murine data are consistent with these predictions. This finding delineates the situation in which there is a linear relationship between dose and provides insight into the limitations of the incubation period assay. Specifically, it suggests that the incubation period assay should be unable to distinguish between different doses below the ID50.

Second, we investigated variability in incubation periods and asked whether TSE incubation periods are highly reproducible. We revealed that they are markedly more variable than predicted by the model. Our findings lead us to question why this is so, especially given that the dose response curve and the mean incubation periods are in close agreement with the model. Could the mechanism of prion growth be incorrect or does the inconsistency lie with data collection or the relationship between prion numbers and clinical symptoms? The most popular model for prion growth, based upon polymer breakage and expansion, is underpinned by exponential growth dynamics and would predict the same variability as seen here. That model therefore also cannot explain the effect that we see. In regards to data collection, some variability is likely to arise from the difficulty in spotting symptoms and from small experimental variability in the dose of the inoculum. It must also be noted that some inaccuracies in the data could arise because the duration of the experiments was finite. At the end of each experiment all surviving mice were culled and examined for the presence of pathological lesion without symptoms might have progressed to disease if the experiments had run for longer. However this does not explain the high variability in incubations periods as if the data were not censored in this way, such unusually long incubation periods would only increase variability, not reduce it.

It is not difficult to propose ways in which prion infection is more complex than a stochastic birth-death process in a homogeneous environment. All the heterogeneities of the in vivo situation: spatial, tissue, temporal and genetic [35] could add to the variability in incubation periods. It is indeed remarkable that such a simple model so clearly reproduces the infection probability and mean incubation period across thousands of mice challenged under such a range of experimental conditions. The intellectual challenge posed by this analysis is to understand what processes are driving the observed variability in incubation periods whilst conserving the infection probabilities and mean incubation periods that are so well explained by the simple model presented here.

## Materials and Methods

### Scrapie titration experiments

The data used in this study were collated from 127 murine scrapie titration experiments, conducted over 30 years (started between 1965 and 1993). In these experiments each mouse was inoculated with tissue from another mouse infected with a mouse-passaged scrapie isolate. Within each experiment, the mice were controlled for mouse breed, scrapie strain, route of inoculation and the tissue from which the inoculum was derived. Between experiments these variables differed such that ten different mouse breeds and seven different scrapie strains were used. The maximum duration of observation also differed between experiments. In the majority of experiments, inoculation was intracranial and the source of infectious material was usually brain; however spleen tissue was occasionally used. In total, 4338 mice were inoculated at varying doses (10 fold dilutions). For each mouse the following information was recorded: 1) whether the animal was killed or died, 2) the number of days between inoculation and death and 3) whether there were clinical signs of scrapie at death. For most mice pathological signs of scrapie were also tested post-mortem. Depending upon the combination of the these factors, each mouse was classified into one of the following categories: 1) uninfected at the end of the experiment (N = 1379), 2) infected with incubation period data (N = 2162), 3) infected without incubation period data (i.e. they died or were killed before clinical symptoms arose but were pathologically positive; N = 57), or 4) died prior to the end of the experiment not of scrapie (N = 740). The precise rules defining these categories, and more details of these experiments are provided in McLean and Bostock [17]. It was highlighted in that report that there is there are no systematic trends that render comparison across the 30 years of experiments invalid. Following estimation of the ID50 for each experiment (explained in Figure S1) eight experiments (195 mice) were removed from further analysis. Data from the remaining 119 experiments are presented in Figure 1 and Table 1.

## Supporting Information

Figure S1 **Calculating the ID50 for each experiment.**
(PDF)

Figure S2 **For each of the six largest experiments, the observed variance of the incubation period is markedly larger than the maximum predicted variance.**
(PDF)

Table S1 Analytic expressions derived from the stochastic model describing the probability of infection and the expectation and variance of incubation period.
(PDF)

Text S1 Derivations for analytic expressions describing the probability of infection and the expectation and variance of the incubation period.
(PDF)
