# Supplementary material for: There Is No Safe Dose of Prions
Source: PLoS One. 2011 Aug 15;6(8):e23664. doi: 10.1371/journal.pone.0023664 (PMC3156228; doi:10.1371/journal.pone.0023664)
Supplement: Figure S1 — Calculating the ID50 for each experiment. (PDF) [file pone.0023664.s001.pdf]

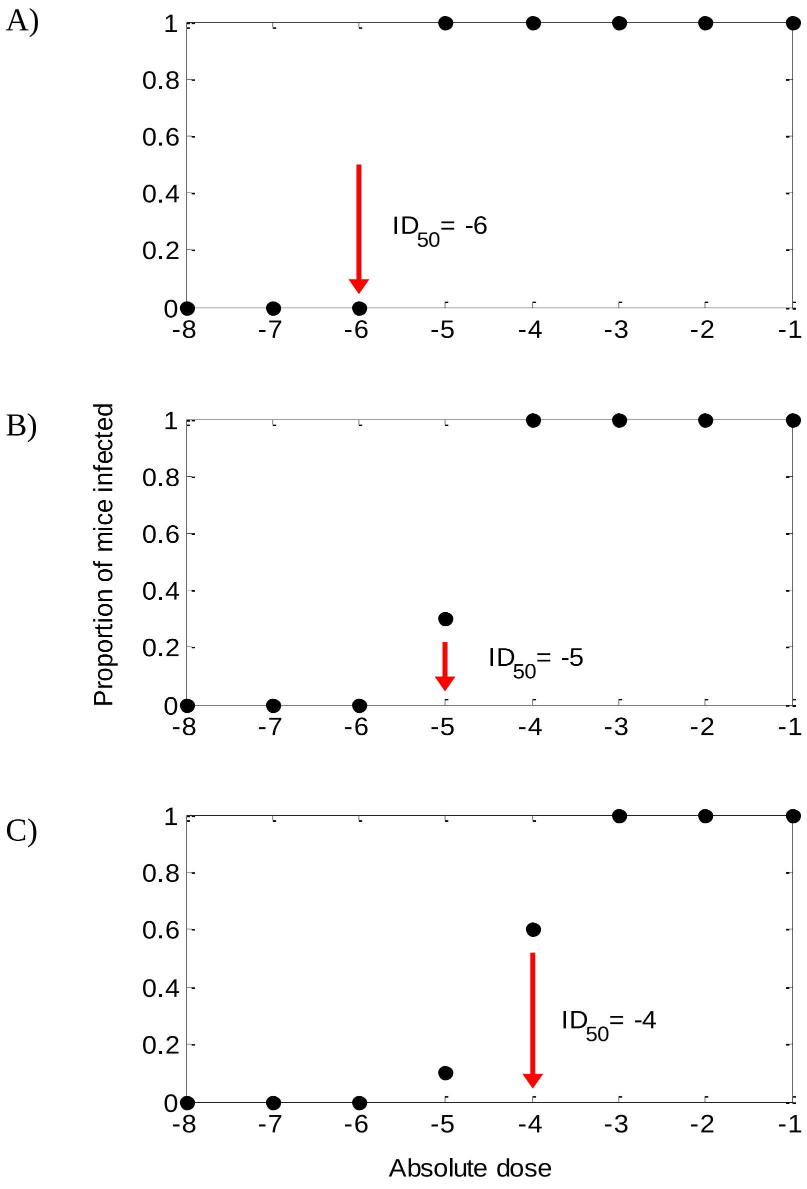

Figure S1. Calculating the ID50 for each experiment.
For each experiment we used data on the proportion of mice infected at each dose (the dose-response data) to estimate the ID50. Firstly, we excluded from further analysis those experiments (N=8) for which fewer than 50% of hosts were infected at each dose or greater than 50% of hosts were infected at each dose. For these experiments it was not possible to accurately estimate the ID50. To estimate the ID50 for the remaining 119 experiments we used three different methods according to the number of doses at which the proportion infected was strictly between zero and one. A) If no dose met this criterion, the last dose with no mice infected was assigned to be the ID50. B) If one dose met this criterion, the dose for that group was assigned to be the ID50. C) If two or more doses met this criterion the ID50 was calculated from the coefficients of a curve fitted by binary logistic regression and rounded to the nearest integer.
