# Supplementary material for: There Is No Safe Dose of Prions
Source: PLoS One. 2011 Aug 15;6(8):e23664. doi: 10.1371/journal.pone.0023664 (PMC3156228; doi:10.1371/journal.pone.0023664)
Supplement: Figure S2 — For each of the six largest experiments, the observed variance of the incubation period is markedly larger than the maximum predicted variance. (PDF) [file pone.0023664.s002.pdf]

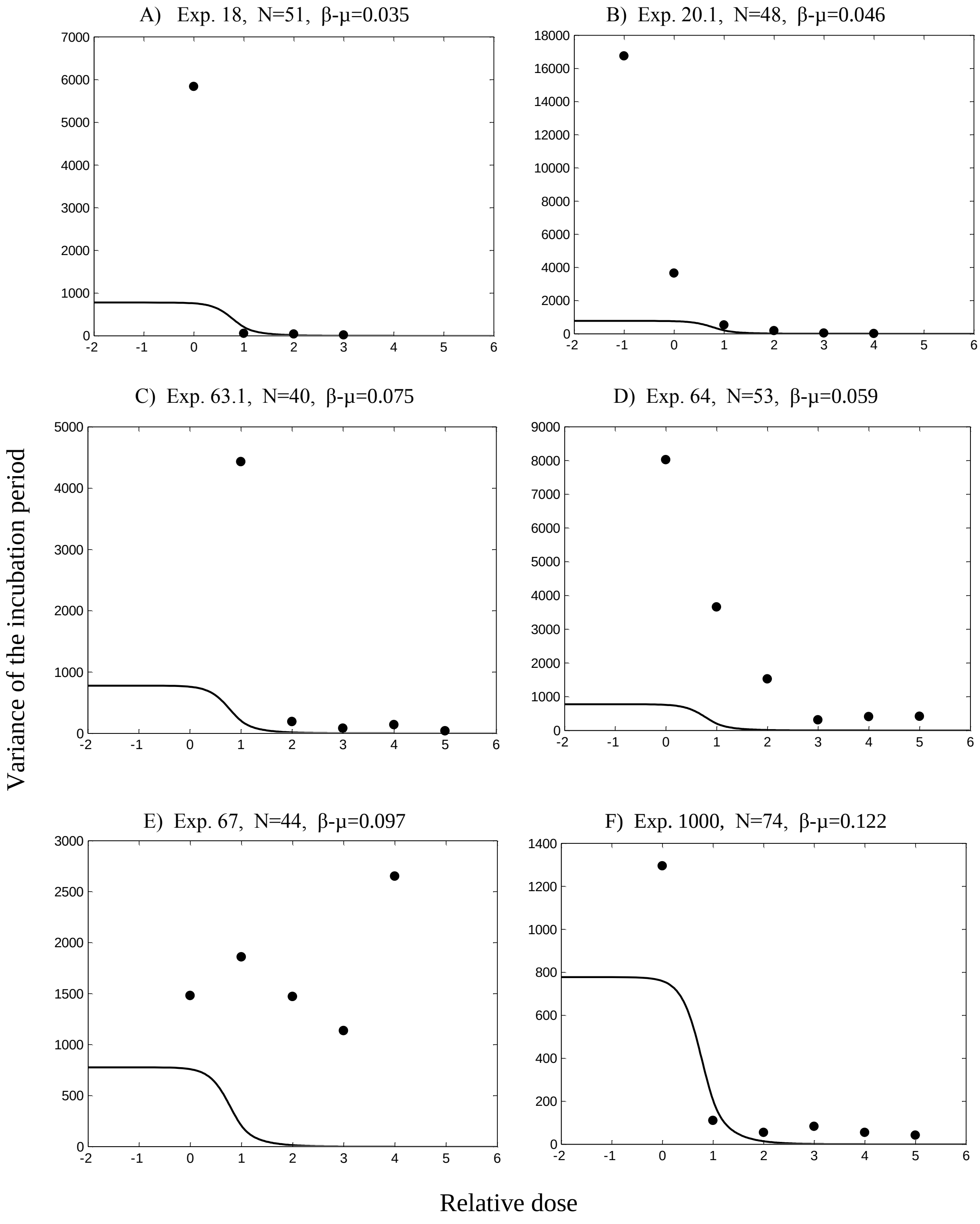

Figure S2. For each of the six largest experiments, the observed variance of the incubation period is markedly larger than the maximum predicted variance.

This figure compares the observed and predicted variance of the incubation period for the six experiments with the largest number of infected mice with an incubation period. For each experiment the observed variance is provided for each relative dose for which there were at least three mice with an incubation period. For each experiment the net growth rate, β-μ, was estimated by fitting the model to the mean incubation periods. Based upon this estimate, a model prediction of the variance over all doses is provided. This comparison shows that for each experiment, the observed variance at low doses is markedly larger than the predicted maximum. The number of mice in each experiment (N) is provided. A) Experiment number 18. B) Experiment number 20.1. C) Experiment number 63.1. D) Experiment number 64. E) Experiment number 67. F) Experiment number 1000.
