# Supplementary material for: There Is No Safe Dose of Prions
Source: PLoS One. 2011 Aug 15;6(8):e23664. doi: 10.1371/journal.pone.0023664 (PMC3156228; doi:10.1371/journal.pone.0023664)
Supplement: Table S1 — Analytic expressions derived from the stochastic model describing the probability of infection and the expectation and variance of incubation period. (PDF) [file pone.0023664.s003.pdf]

Function	Expression
Probability of infection, p	$p = \frac{(\mu/\beta)^x - 1}{(\mu/\beta)^L - 1} = 1 - 0.5^{10^{RD}}$
Expected incubation period, $E(T)$	$E(T) = \sum_{n=x}^{L-1} \Omega_n$ Where $\Omega_n = \frac{1}{(\beta^{n+1} - \mu^{n+1})}\left(\frac{\beta^n - \mu^n}{n} + \mu\beta(\beta^{n-1} - \mu^{n-1})\Omega_{n-1}\right)$ For positive relative doses, this can be approximated to: $E(T) = \frac{1}{\beta - \mu}\ln\left(\frac{L}{x}\right) = \frac{\ln(10)}{\beta - \mu}(\mathrm{RD_L} - \mathrm{RD})$
Variance of the incubation period, $Var(T)$	$Var(T) = \sum_{n=x}^{L-1} \Lambda_n$ Where: $\Lambda_n = \frac{(\beta^n - \mu^n)}{n^2(\beta+\mu)(\beta^{n+1} - \mu^{n+1})} + \beta\mu(\beta^{n-1} - \mu^{n-1})\left(\frac{\Lambda_{n-1}}{(\beta^{n+1} - \mu^{n+1})} + \frac{(\Omega_{n-1} + \Omega_n)^2}{(\beta+\mu)(\beta^n - \mu^n)}\right)$

Table S1. Analytic expressions derived from the stochastic model describing the probability of infection and the expectation and variance of incubation period.

Analytic expressions derived from the stochastic model describing how the probability of infection and the mean and variance of the incubation period change with the model parameters. These expressions are used to plot the model predictions shown in Figures 3 and S3. In the model a mouse is inoculated with a dose of *x* prions. The number of prions then changes according to a stochastic birth-death process in which prions are created at rate *β* per prion and die at rate *μ* per prion. The probability of infection, *p*, represents the probability that the number of prions reaches the disease limit, *L*, before it reaches zero. The incubation period, *T*, represents the time it takes for the number of prions to multiply from the inoculating dose, *x*, to the disease limit, *L*. RD represents the relative dose and $\mathrm{RD_L}$ represents the relative dose at the disease limit. The derivation of these expressions is provided in Text S1.
