# Supplementary material for: There Is No Safe Dose of Prions
Source: PLoS One. 2011 Aug 15;6(8):e23664. doi: 10.1371/journal.pone.0023664 (PMC3156228; doi:10.1371/journal.pone.0023664)
Supplement: Text S1 — Derivations for analytic expressions describing the probability of infection and the expectation and variance of the incubation period. (PDF) [file pone.0023664.s004.pdf]

Text S1. Derivations for analytic expressions describing the probability of infection and the expectation and variance of the incubation period.

Here we present derivations for the analytic expression presented in Table S1. These expressions describe the probability of infection and the expectation and variance of the incubation period in the stochastic model of the replication of prions. A host (a mouse) is inoculated with a dose of *x* prions. Prion numbers then change according to a stochastic process until the number of prions reaches 0 (grey jagged line Figure 2B) or the disease limit, L (black jagged line, Figure 2B). For those hosts in whom the disease dose is reached, the time taken to reach this dose is defined as the incubation period. Traditionally, this mathematical problem is described a linear birth-death process with two absorbing barriers (at 0 and L) and birth and death rates proportional to the population size. Since we are only interested in the case where infection is possible, we specify that the birth rate is greater than the death rate ($\beta > \mu$). The variables and parameters used to derive the expressions are provided in the list below.

Variables	
t	Time.
n	The number of prions.
n^m	The number of prions after *m* events.
$n(t)$	The number of prions at time *t*.
Q_n	The time, starting from *n* prions, it takes before there are $n+1$ prions given that the host becomes infected.
T	The incubation period, i.e. the time, starting from *x* prions, it takes for the process to reach *L* prions.
I_n	An indicator variable defining whether the first transition from state *n* (*n* prions) is a birth ($I_n = 1$) or a death ($I_n = 0$).
G_n	An indicator variable defining whether the first transition from state *n* given that the host becomes infected is a birth ($G_n = 1$) or a death ($G_n = 0$).
F_n	The event that the host becomes infected (i.e. that the number of prions reaches *L* before it reaches 0), given a starting dose of *n* prions.
p_n	The probability of infection, starting at a dose of *n* prions ($P(F_n)$).
X_n	The time to the next event when there are *n* prions.
RD	Relative dose $= \log_{10}\left(x/\text{ID50}\right)$.
Ω_n	The expectation of Q_n ($= E(\Omega_n)$).
Λ_n	The variance of Q_n ($= Var(\Omega_n)$).
Parameters	
μ	Death rate of prions (years^{-1}).
β	Birth rate of prions (years^{-1}).
x	Number of prions in the inoculating dose.
L	The number of prions at the disease limit.
ID50	Infectious dose 50%.
RD_L	Relative dose at the disease limit $= \log_{10}\left(L/\text{ID50}\right)$.

1. The probability of infection, p

To find an expression for the probability of infection we consider how the number of prions will change from the start of the infection. At the start of the infection there are *x* prions. Define an event as either a birth of a prion or a death of a prion. Then the change in the number of prions can be regarded as a random walk consisting of a series of these events and where the number of prions after each event increases by 1 if the event is a birth or decreases by 1 if the event is a death. The random walk ends when the number of prions reaches zero or the disease limit (*L*). We define n^m be the number of prions after the mth event, thus, more specifically, n^m is a random walk on {0,1,…L with absorbing barriers at 0 and L. The starting value is $n^0 = x$. To find the probability of infection we simply need to find the probability that the random walk reaches the disease limit ($n^m = L$) before it reaches zero ($n^m = 0$). Part of this proof is provided in Grimmett and Welsh 1986 [22] where it is described in the context of the 'Gambler's Ruin' problem.

Since each transition in the random walk can be either a birth or a death, the probabilities assigned to the random walk are given by:

$$n^m = \begin{cases} n^{m-1}+1 \text{ (birth)} & \text{with probability } \beta/(\beta+\mu) \\ n^{m-1}-1 \text{ (death)} & \text{with probability } \mu/(\beta+\mu) \end{cases} \qquad \textbf{(1)}$$

Let I_n be an indicator variable defining whether the first transition from state n is to state $n+1$ ($I_n = 1$) or to state $n-1$ ($I_n = 0$). Also let F_x be the event that the host becomes infected, given a starting dose of *x* prions, i.e. the event that the number of prions reaches *L* before it reaches 0.

Using the partition theorem we can write:

$$P(F_x) = P(F_x|I_1{=}1)P(I_1{=}1) + P(F_x|\, I_1{=}0)P(I_1{=}0) \qquad \textbf{(2)}$$

$$\Rightarrow P(F_x) = P(F_x|\text{start at dose } x{+}1)P(I_1{=}1) + P(F_x|\text{ start at dose } x\text{-}1)P(I_1{=}0) \qquad \textbf{(3)}$$

$$\Rightarrow P(F_x) = P(F_{x+1})P(I_1{=}1) + P(F_{x-1})P(I_1{=}0) \qquad \textbf{(4)}$$

Let $p_x = P(F_x) =$ the probability of infection starting at dose *x.* Since $P(I^1{=}1) = P(n^1 = x+1) = \beta/(\beta+\mu)$ and $P(I^1{=}1) = P(n^1 = x-1) = \mu/(\beta+\mu)$.

$$\Rightarrow \quad p_x = p_{x+1}\left(\frac{\beta}{\beta+\mu}\right) + p_{x-1}\left(\frac{\mu}{\beta+\mu}\right) \quad \text{for } 1 \le x \le L-1 \qquad \textbf{(5)}$$

This is a homogeneous second order difference equation with boundary conditions $p_0 = 0$ and $p_L = 1$. The general solution of this equation for $\mu \ne \beta$ is given by:

$$\boxed{p_x = \frac{(\mu/\beta)^x - 1}{(\mu/\beta)^L - 1}} \qquad \textbf{(6)}$$

Since $\beta > \mu$, and since *L* is large we substitute into equation 6 the approximation $(\mu/\beta)^L = 0$.

$$p_x = 1 - (\mu/\beta)^x \qquad \textbf{(7)}$$

Note that an expression for the ID50 can be found by substituting into equation 7, $p_{\text{ID50}} = 0.5$ and $x = \text{ID50}$.

$$0.5 = 1 - (\mu/\beta)^{\text{ID50}} \qquad \textbf{(8)}$$

$$\left(\mu/\beta\right) = 0.5^{\frac{1}{\mathrm{ID50}}} \quad \textbf{(9)}$$

$$p_x = 1 - 0.5^{\frac{x}{\mathrm{ID50}}} \quad \textbf{(10)}$$

We define the relative dose (RD) to be: $\mathrm{RD} = \log_{10}\left(\frac{x}{\mathrm{ID50}}\right)$. Therefore the probability of infection (which we rename as *p*) is given by the formula below. It is dependent only upon the relative dose.

$$\boxed{p = 1 - 0.5^{10^{RD}}} \quad \textbf{(11)}$$

Equation 10 can be linearized to show that the probability of infection is proportional to the dose ($p_x \propto x$) at low doses (i.e. when $x \ll \mathrm{ID50}$).

$$p_x = 1 - 0.5^{\frac{x}{\mathrm{ID50}}} = 1 - \left(1 + \frac{x\ln(0.5)}{ID50} - \frac{1}{2!}\left(\frac{x\ln(0.5)}{ID50}\right)^2 + \frac{1}{3!}\left(\frac{x\ln(0.5)}{ID50}\right)^3 + \ldots\right) \approx -\frac{x\ln(0.5)}{\mathrm{ID50}} \propto x \quad \textbf{(12)}$$

2. The expectation of the incubation period, *E(T).*

We describe how to find an expression for the expectation of the incubation period. The first thing to note is that where in the derivation for the probability of infection we considered a random walk where the dummy variable *m* represented the number of events that have taken place, in this proof we introduce a continuous measure of time since inoculation *t* (e.g. days). We define the incubation period *T* to be the time between inoculation and infection. In relation to the model, this is the time it takes for the number of prions in the population to grow from the inoculating dose (*x*) to the disease limit (*L*). Note that this time is naturally conditioned on infection, i.e. on the number of prions reaching *L* before 0. The derivation we provide here is adapted from one provided in Ross (1997) [34].

Let Q_n (for $n \geq 1$) be the time, starting from state *n* (*n* prions), it takes for state *n*+1 to first be reached given that the host becomes infected. For example, Q_3 is the time, starting from state 10 (10 prions in population), it takes for the process to first enter state 11 (11 prions in the population) given that the host becomes infected.

The incubation period, *T*, is simply Q_n summed from the inoculating dose (*x*) to *L-1*.

$$T = \sum_{n=x}^{L-1} Q_n \quad \textbf{(13)}$$

We aim to find an expression for the expectation of the incubation period ($E(T)$). Since this can be written as the sum of the expectations of the Q_n's, we will recursively compute $E(Q_n)$ for $n = x$ to $L-1$.

$$E(T) = \sum_{n=x}^{L-1} E(Q_n) \quad \textbf{(14)}$$

We assume that the birth-death process presented is a stochastic Poisson process, i.e. events (births and deaths of prions) occur continuously and independently of one another. Thus, the time between events are described by exponential distributions. More specifically, if we define X_n to be the time until the next event when there are *n* prions in the population then X_n is an exponential distribution with rate $n(\beta + \mu)$, i.e. the rate is proportional to the product of the number of prions in the population and the sum of the birth and death rate.

$$X_n \sim Exp\left(n(\beta+\mu)\right) \quad \textbf{(15)}$$

As defined above, Q_1 is the time, starting from 1 prion it takes before there are 2 prions given that the host becomes infected. Since the random walk needs to go directly from 1 prion to 2 prions to avoid reaching 0, Q_1 is simply the time to the next event when there 1 prion in the population. Therefore:

$$Q_1 \sim X_1 \sim Exp(\beta+\mu) \quad \textbf{(16)}$$

And

$$E(Q_1) = \frac{1}{\beta+\mu} \quad \textbf{(17)}$$

Define $G_n \sim I_n \mid F_n$, i.e.

$$G_n = \begin{cases} 1 & \text{if the 1st transition from } n \text{ is to } n+1 \text{ (a birth), given the host becomes infected} \\ 0 & \text{if the 1st transition from } n \text{ is to } n-1 \text{ (a death), given the host becomes infected} \end{cases} \quad \textbf{(18)}$$

To calculate $E(Q_n)$ for $n \geq 2$ we can condition on whether the first transition from *n* is a birth given the host becomes infected starting from dose n ($G_n = 1$) or whether the first transition from *n* is a death given that the host becomes infected from dose *n* ($G_n = 0$):

$$E(Q_n) = E\left(Q_n \mid G_n = 1\right)P(G_n = 1) + E\left(Q_n \mid G_n = 0\right)P(G_n = 0) \quad \textbf{(19)}$$

To calculate $E(Q_n \mid G_n = 1)$ and $E(Q_n \mid G_n = 0)$ note that, independent on whether the first transition is a birth or death, the time until it occurs, X_n, is exponential with rate $n(\beta+\mu)$. If this first transition is a birth the population size is $n+1$, so no additional time is needed; whereas if it's a death, then the population size becomes $n-1$ and the additional time needed to reach $n+1$ is equal to the time it takes to return to state *n* (this has mean $E(Q_{n-1})$) plus the additional time it takes to reach $n+1$ (this has mean $E(Q_n)$). Thus:

$$E\left(Q_n \mid G_n = 1\right) = \frac{1}{n(\beta+\mu)} \quad \textbf{(20)}$$

$$E\left(Q_n \mid G_n = 0\right) = \frac{1}{n(\beta+\mu)} + E(Q_{n-1}) + E(Q_n) \quad \textbf{(21)}$$

To calculate $P(G_n = 1)$ and $P(G_n = 0)$ we use a standard conditional probability formulae:

$$P(G_n = 1) = P(I_n = 1 \mid F_n) = \frac{P(F_n \mid I_n = 1)P(I_n = 1)}{P(F_n)} = \frac{p_{n+1}P(I_n = 1)}{p_n} = \frac{p_{n+1}\beta}{p_n(\beta+\mu)} \quad \textbf{(22)}$$

$$P(G_n = 0) = P(I_n = 0 \mid F_n) = \frac{P(F_n \mid I_n = 0)P(I_n = 0)}{P(F_n)} = \frac{p_{n-1}P(I_n = 0)}{p_n} = \frac{p_{n-1}\mu}{p_n(\beta+\mu)} \quad \textbf{(23)}$$

By substituting equations 20-23 into equation 19, we find:

$$E(Q_n) = \frac{1}{n(\beta+\mu)} \frac{p_{n+1}}{p_n} \frac{\beta}{(\beta+\mu)} + \left(\frac{1}{n(\beta+\mu)} + E(Q_{n-1}) + E(Q_n)\right) \frac{p_{n-1}}{p_n} \frac{\mu}{(\beta+\mu)} \quad \textbf{(24)}$$

By substituting in the expression for p_n (equation 6) and solving for $E(Q_n)$ we find:

$$E(Q_n) = \frac{1}{\left(\mu^{n+1} - \beta^{n+1}\right)} \left(\frac{\mu^n - \beta^n}{n} + \mu\beta(\mu^{n-1} - \beta^{n-1}) E(Q_{n-1}) \right) \quad \textbf{(25)}$$

Define Ω_n to be the expectation of Q_n $(\Omega_n = E(Q_n))$, then equations 14 and 25 can be rewritten as:

$$\boxed{E(T) = \sum_{n=x}^{L-1} \Omega_n} \quad \textbf{(26)}$$

$$\boxed{\Omega_n = \frac{1}{\left(\mu^{n+1} - \beta^{n+1}\right)} \left(\frac{\mu^n - \beta^n}{n} + \mu\beta(\mu^{n-1} - \beta^{n-1}) \Omega_{n-1} \right)} \quad \textbf{(27)}$$

As doses increase beyond the ID50 (relative dose 0) the probability of infection quickly approaches 1 (see Figure 1A). For positive relative doses the following approximation to $E(Q_n)$ can therefore be found by substituting $p_{n+1} = p_n = p_{n-1} = 1$ into equation 24.

$$E(Q_n) = \frac{1}{n\beta} + \frac{\mu}{\beta} E(Q_{n-1}) \quad \textbf{(28)}$$

After substituting this expression into equation 14 and using the iterative method it can be shown that for large relative doses:

$$\boxed{E(T) \approx \frac{1}{\beta - \mu} \ln\left(\frac{L}{x}\right)} \quad \textbf{(29)}$$

The relative dose is defined as: $\mathrm{RD} = \log_{10}\left(\frac{x}{\mathrm{ID50}}\right)$ and the relative dose at the disease limit can be defined as $\mathrm{RD_L} = \log_{10}\left(\frac{L}{\mathrm{ID50}}\right)$. Therefore the expectation of the incubation period beyond approximately relative dose 0 can be written in terms of the relative dose (RD), the relative dose at the disease limit ($\mathrm{RD_L}$) and the net growth rate (β-μ). The expression shows that the expectation of the incubation period is:

$$\boxed{E(T) \approx \frac{\ln(10)}{\beta - \mu} \left(\mathrm{RD_L} - \mathrm{RD}\right)} \quad \textbf{(30)}$$

Thus for positive relative doses the expectation of the incubation period decreases linearly with relative dose.

3. The variance of the incubation period, Var(T)

We aim to find the variance of the incubation period, $Var(T)$. This proof is adapted from one provided in Ross (1997) [34]. Since $T = \sum_{n=x}^{L-1} Q_n$ (equation 13) and since, by the Markovian property, the successive random variables (Q_n) are independent, it follows that:

$$Var(T) = \sum_{n=x}^{L-1} Var(Q_n) \quad \textbf{(31)}$$

Therefore we need to compute $Var(Q_n)$ for each n. These can be computed recursively starting at $Var(Q_1)$. Since $Q_1 \sim X_1 \sim Exp(\beta+\mu)$ (equation 16)

$$Var(Q_1) = \frac{1}{(\beta+\mu)^2} \quad \textbf{(32)}$$

To find $Var(Q_n)$ for $n \geq 2$, we can use the conditional variance formulae on the two random variables, Q_n and G_n

$$Var(Q_n) = Var\left(E(Q_n \mid G_n)\right) + E\left(Var(Q_n \mid G_n)\right) \quad \textbf{(33)}$$

<u>First find</u> $Var\left(E(Q_n \mid G_n)\right)$

Note that equations 20 and 21 can be written as:

$$E(Q_n \mid G_n) = \frac{1}{(\beta+\mu)n} + (1-G_n)\left(E(Q_{n-1}) + E(Q_n)\right) \quad \textbf{(34)}$$

$$\Rightarrow Var\left(E(Q_n \mid G_n)\right) = Var(G_n)\left(E(Q_{n-1}) + E(Q_n)\right)^2 \quad \textbf{(35)}$$

Find $Var(G_n)$:

$$Var(G_n) = E\left(G_n{}^2\right) - \left(E(G_n)\right)^2 \quad \textbf{(36)}$$

From equations 22 and 23 we have:

$$P(G_n = 1) = \frac{p_{n+1}\beta}{p_n(\beta+\mu)} \quad \textbf{(37)}$$

$$P(G_n = 0) = \frac{p_{n-1}\mu}{p_n(\beta+\mu)} \quad \textbf{(38)}$$

Therefore

$$E(G_n) = 1 \times P(G_n = 1) + 0 \times P(G_n = 0) = \frac{p_{n+1}\beta}{p_n(\beta+\mu)} \quad \textbf{(39)}$$

And

$$E(G_n{}^2) = 1^2 \times P(G_n = 1) + 0^2 \times P(G_n = 0) = \frac{p_{n+1}\beta}{p_n(\beta+\mu)} \quad \textbf{(40)}$$

$$\Rightarrow Var(G_n) = \frac{p_{n+1}\beta}{p_n(\beta+\mu)}\left(1 - \frac{p_{n+1}\beta}{p_n(\beta+\mu)}\right) \quad \textbf{(41)}$$

$$\begin{aligned}\Rightarrow Var\left(E(Q_n \mid G_n)\right) &= Var(G_n)\left(E(Q_{n-1}) + E(Q_n)\right)^2 \\ &= \frac{\beta p_{n+1}}{(\beta+\mu)p_n}\left(1 - \frac{\beta p_{n+1}}{(\beta+\mu)p_n}\right)\left(E(Q_{n-1}) + E(Q_n)\right)^2\end{aligned} \quad \textbf{(42)}$$

<u>Second find</u> $E\left(Var(Q_n \mid G_n)\right)$

As described previously, X_n = the time until the next event when there are *n* prions in the population. $X_n \sim Exp\left(n(\beta+\mu)\right)$

First note that : $\left(Q_n \mid G_n = 1\right) \sim \left(X_n \mid G_n = 1\right)$

$$\Rightarrow Var(Q_n \mid G_n = 1) = Var(X_n \mid G_n = 1) = Var(X_n) \quad \textbf{(43)}$$

And:

$$Var(Q_n \mid I_n = 0) = Var(X_n + \text{ time to get back to } n + \text{ time to then reach n+1}) \quad \textbf{(44)}$$
$$= Var(X_n) + Var(Q_{n-1}) + Var(Q_n)$$

Since X_n, Q_{n-1} and Q_n are independent random variables and since $Var(X_n) = \frac{1}{n^2(\beta+\mu)^2}$

$$\Rightarrow Var(Q_n \mid G_n) = Var(X_n) + (1-G_n)\left(Var(Q_{n-1}) + Var(Q_n)\right) \quad \textbf{(45)}$$

$$\Rightarrow E\left[Var(Q_n \mid G_n)\right] = \frac{1}{n^2(\beta+\mu)^2} + \left(1 - E(G_n)\right)\left(Var(Q_{n-1}) + Var(Q_n)\right) \quad \textbf{(46)}$$

$$\Rightarrow E\left[Var(Q_n \mid G_n)\right] = \frac{1}{n^2(\beta+\mu)^2} + \left(1 - \frac{\beta p_{n+1}}{(\beta+\mu)p_n}\right)\left(Var(Q_{n-1}) + Var(Q_n)\right) \quad \textbf{(47)}$$

Substitute equations 42 and 47 into equation 33:

$$Var(Q_n) = \frac{1}{n^2(\beta+\mu)^2} + \left(1 - \frac{\beta p_{n+1}}{(\beta+\mu)p_n}\right)\left(Var(Q_{n-1}) + Var(Q_n)\right)$$
$$+ \frac{\beta p_{n+1}}{(\beta+\mu)p_n}\left(1 - \frac{\beta p_{n+1}}{(\beta+\mu)p_n}\right)\left(E(Q_{n-1}) + E(Q_n)\right)^2 \quad \textbf{(48)}$$

Substitute in the expression for p_n (equation 6) and solve for $Var(Q_n)$:

$$Var(Q_n) = \frac{(\mu^n - \beta^n)}{n^2\left(\beta+\mu\right)(\mu^{n+1} - \beta^{n+1})} + \beta\mu(\mu^{n-1} - \beta^{n-1})\left(\frac{Var(Q_{n-1})}{(\mu^{n+1} - \beta^{n+1})} + \frac{\left(E(Q_{n-1}) + E(Q_n)\right)^2}{(\beta+\mu)(\mu^n - \beta^n)}\right) \quad \textbf{(49)}$$

Define Λ_n to be the variance of Q_n ($\Lambda_n = Var(Q_n)$) therefore equations 14 and 49 can be rewritten as:

$$\boxed{Var\left(T\right) = \sum_{n=x}^{L-1} \Lambda_n} \quad \textbf{(50)}$$

$$\boxed{\Lambda_n = \frac{(\beta^n - \mu^n)}{n^2\left(\beta+\mu\right)(\beta^{n+1} - \mu^{n+1})} + \beta\mu(\beta^{n-1} - \mu^{n-1})\left(\frac{\Lambda_{n-1}}{(\beta^{n+1} - \mu^{n+1})} + \frac{\left(\Omega_{n-1} + \Omega_n\right)^2}{(\beta+\mu)(\beta^n - \mu^n)}\right)} \quad \textbf{(51)}$$
